# Discovery of Diverse Small Molecule Chemotypes with Cell-Based PKD1 Inhibitory Activity

**DOI:** 10.1371/journal.pone.0025134

**Published:** 2011-10-05

**Authors:** Elizabeth R. Sharlow, Gabriela Mustata Wilson, David Close, Stephanie Leimgruber, Manuj Tandon, Robyn B. Reed, Tong Ying Shun, Q. Jane Wang, Peter Wipf, John S. Lazo

**Affiliations:** 1 Department of Pharmacology, University of Virginia, Charlottesville, Virginia, United States of America; 2 Department of Chemistry, University of Virginia, Charlottesville, Virginia, United States of America; 3 Department of Computational and Systems Biology, University of Pittsburgh, Pittsburgh, Pennsylvania, United States of America; 4 Drug Discovery Institute, University of Pittsburgh, Pittsburgh, Pennsylvania, United States of America; 5 Department of Pharmacology and Chemical Biology, University of Pittsburgh, Pittsburgh, Pennsylvania, United States of America; 6 Department of Chemistry, University of Pittsburgh, Pittsburgh, Pennsylvania, United States of America; Stanford University, United States of America

## Abstract

Protein kinase D (PKD) is a novel family of serine/threonine kinases regulated by diacylglycerol, which is involved in multiple cellular processes and various pathological conditions. The limited number of cell-active, selective inhibitors has historically restricted biochemical and pharmacological studies of PKD. We now markedly expand the PKD1 inhibitory chemotype inventory with eleven additional novel small molecule PKD1 inhibitors derived from our high throughput screening campaigns. The *in vitro* IC_50_s for these eleven compounds ranged in potency from 0.4 to 6.1 µM with all of the evaluated compounds being competitive with ATP. Three of the inhibitors (CID 1893668, (1Z)-1-(3-ethyl-5-methoxy-1,3-benzothiazol-2-ylidene)propan-2-one; CID 2011756, 5-(3-chlorophenyl)-N-[4-(morpholin-4-ylmethyl)phenyl]furan-2-carboxamide; CID 5389142, (6Z)-6-[4-(3-aminopropylamino)-6-methyl-1H-pyrimidin-2-ylidene]cyclohexa-2,4-dien-1-one) inhibited phorbol ester-induced endogenous PKD1 activation in LNCaP prostate cancer cells in a concentration-dependent manner. The specificity of these compounds for PKD1 inhibitory activity was supported by kinase assay counter screens as well as by bioinformatics searches. Moreover, computational analyses of these novel cell-active PKD1 inhibitors indicated that they were structurally distinct from the previously described cell-active PKD1 inhibitors while computational docking of the new cell-active compounds in a highly conserved ATP-binding cleft suggests opportunities for structural modification. In summary, we have discovered novel PKD1 inhibitors with *in vitro* and cell-based inhibitory activity, thus successfully expanding the structural diversity of small molecule inhibitors available for this important pharmacological target.

## Introduction

Protein kinase D1 (PKD1/PKCμ; GenBank: ABE96833.1) is a member of a novel family of serine/threonine kinases characterized by their diacylglycerol-dependent regulation. PKD1 is one of three PKD isoforms (PKD1–3), which have well recognized roles in cell proliferation, survival, invasion and protein transport [1]. Although functional redundancy has been documented among PKD1–3, evidence suggests specialized roles for each isoform, most likely due to differences in protein structure as well as expression patterns, protein localization and substrate proximity [2], [3], [4], [5], [6]. Extensive studies have documented a role of PKD proteins in cancer and cardiac cell model systems; however, considerable evidence supports roles for PKDs in neuronal signaling pathways, DNA damage, growth factor signaling, embryogenesis, multi-drug resistance, metabolic disorders, inflammation and immune responses, further emphasizing the critical role of PKDs across multiple biological systems [7], [8], [9], [10], [11], [12]. Although loss of PKD function experiments (*i.e.*, dominant negative mutants, antisense oligonucleotides and RNA interference) have been used to characterize the functional role of PKDs in various model systems, studies had been hampered by the lack of readily available potent and PKD specific/selective small molecule inhibitors [13], [14], [15]. The lack of structural data for PKDs hinder our ability to exploit traditional structure-based drug design approaches to expedite the identification of more potent and highly specific PKD inhibitors. Nevertheless, previous comparative studies of the kinase family using sequence similarity revealed that kinases share a conserved catalytic core where ATP and substrate binding and phosphate transfer occurs [16]. Moreover, Thompson *et al.* [17] reported the three-dimensional structure of the catalytic domain of the protein serine/threonine kinase, cAMP-dependent protein kinase (PKA) and compared it to a set of 10 active kinase crystal structures spanning the kinome. Using a unique pocket clustering methodology, they found there is little deviation with respect to the spatial conservation within kinase active site clefts [17]. Thus, this model may be exploitable to help characterize ATP competitive kinase inhibitors in the absence of crystal structures.

We previously identified and characterized the first selective *in vitro* PKD1 inhibitor with cellular activity: CID 755673 [18]. This compound is a non-ATP competitive, pan-PKD inhibitor discovered through implementation of *in vitro* immobilized metal affinity for phosphochemicals (IMAP) PKD1 fluorescence polarization high throughput screening (HTS) assay [18], [19]. Using CID 755673 as a parental structure, subsequent analogue development resulted in PKD1 inhibitors with increased potency in both *in vitro* and cell-based models [20], [21]. Additional compounds, also initially identified by others from *in vitro* HTS assays, have been reported as PKD inhibitors, including CRT5 as well as novel 3,5-diarylazole and 2,6-naphthyridine compounds [22], [23], [24], [25], [26], [27]. Similar to CID 755673, these compounds are pan-PKD small molecule inhibitors with cellular inhibitory activity. Moreover, subsequent chemical modifications of these parental chemotypes have improved their *in vitro* and cellular potency and specificity, yielding, for example, the PKD inhibitors BPKDi and CRT0066101 [25], [28]. However, direct comparison of these latter PKD inhibitors is limited, as precise structural information is not available for CRT0066101. Currently, it appears that all reported PKD small molecule inhibitors have some form of liability, including physicochemical (*i.e.*, poor potency or specificity), off-target effects or limited availability to the scientific community.

Herein, we describe 11 novel PKD1 inhibitory chemotypes identified through our previous HTS campaigns. All compounds displayed *in vitro* PKD1 activity in two independent *in vitro* assay formats and were characterized with a series of secondary assays. Three novel chemotypes inhibited phorbol ester-induced endogenous PKD1 activation (*i.e.*, PKD Ser^916^ phosphorylation) in LNCaP prostate cancer cells and were structurally dissimilar to CID 755673, BKPDi and CRT5, revealing an expanded diversity of PKD1 small molecule inhibitors.

## Materials and Methods

### Chemicals and Reagents

Black opaque small volume microtiter assay plates were purchased from Greiner (Monroe, NC) and used for all immobilized metal affinity for phosphochemicals (IMAP™)-based fluorescence polarization (FP) assay development, primary HTS and secondary confirmation studies. IMAP progressive binding reagent, binding buffer (∼pH 5.5), kinase reaction buffer (10 mM Tris-HCl, pH 7.2, 10 mM MgCl_2_, 0.05% NaN_3_, 1 mM DTT and 0.1% bovine serum albumin) and substrate peptide (FAM-KKLNRTLSVA) were obtained from Molecular Devices (Sunnyvale, CA). Kinase active His-tagged recombinant human protein kinase D1 (PKD1) was obtained from Millipore (Billerica, MA). ATP was purchased from GE Healthcare (Piscataway, NJ). H-89 and Gö6976 were obtained from Millipore and Calbiochem, respectively. DMSO, Syntide-2 substrate peptide and the Library of Pharmacologically Active Compounds (LOPAC/1280 compounds) set were purchased from Sigma-Aldrich (St. Louis, MO). The 196,173 compound library screened for PKD1 small molecule inhibitors in the IMAP FP HTS assay was made available by the Pittsburgh Molecular Library Screening Center (PMLSC; Pittsburgh, PA) as part of the NIH Molecular Library Screening Center Network Roadmap Initiative (Pilot phase). Specifically, this is diversity-based compound library obtained via the NIH Molecular Libraries Small Molecule Repository which collects compounds for HTS and distributed them for use by the NIH Molecular Libraries network-designated screening Centers. Compounds selected from the PMLSC library for further analysis were supplied by Biofocus DPI (A Galapagos Company, San Francisco, CA). After initial IMAP assay IC_50_ confirmation studies, parental compounds were purchased from the commercial vendors ChemDiv (PubChem CID 755673, 646236)(San Diego, CA); Chembridge (PubChem CID 2876479, 2958734)(San Diego, CA); InterBioScreen (PubChem CID 5389142, 663844, 2011756) (Moscow, Russia); Nanosyn (PubChem CID 4438738 (Menlo Park, CA); VWR (PubChem CID 16230, 9549170) (West Chester, PA); Santa Cruz Biotechnology (PubChem CID 1893668/TG003) (Santa Cruz, CA); and Specs-AG (PubChem CID 5086667) (Delft, Netherlands).

### IMAP-based PKD1 FP high throughput screening assay implementation

An HTS IMAP-based PKD1 FP assay was developed and used to screen the PMLSC library to identify primary active compounds as previously described [18], [19]. In brief, the basis for the *in vitro* homogeneous IMAP PKD1 FP assay is the interaction of nanoparticles with covalent phosphorylated moieties generated when active PKD1 enzyme phosphorylates small, fluorescently labeled substrate peptides. The PKD1 kinase reactions were assembled stepwise by the addition of 3× substrate/ATP (300 nM/60 µM), compound and PKD1 enzyme (0.18 units/mL). Positive (MAX) and negative (MIN) control kinase reactions contained DMSO (1%) and Gö6976 (1 µM) or H-89 (100 µM) in 1% DMSO (final concentrations), respectively. All assay plates were setup in a standard 384-well configuration as previously described and all reagents were prepared in kinase reaction buffer [19]. Kinase reactions were incubated for 90 min at room temperature and then were stopped with the addition of 18 µL of IMAP binding reagents (1∶400) in 1× binding buffer A (proprietary composition, Molecular Devices). These nanoparticles associate with substrate peptide phospho groups creating a larger phosphosubstrate–nanoparticle complex. The phosphosubstrate–nanoparticle binding can be quantified and is expressed as millipolarization (mP) units. Inhibition of phosphosubstrate-nanoparticle formation is reflected in lower mP signals which suggested a potential inhibition of PKD1 enzymatic activity. Assay plates were centrifuged at 50 g for 1 min, incubated at room temperature for 2 hr and FP data were collected on a Molecular Devices SpectraMax M5 (excitation A_485_/emission A_525_). This assay was also used in secondary concentration response studies to initially determine IC_50_ concentrations of compounds. IC_50_ determinations were performed using GraphPad Prism software 5.0 (La Jolla, CA).

Compounds were also screened for interference with the IMAP FP assay format. Briefly, PKD1 kinase reactions were allowed to proceed in the absence of compounds as described above. Compounds (final concentration 10 µM) were then added to the kinase reactions followed by the IMAP binding reagent to each microtiter plate well. FP data were collected as described above. Compounds exhibiting >25% inhibition of the signal were considered to interfere with the IMAP FP assay format.

### *In vitro* radiometric PKD assays

For compound IC_50_ confirmation studies, 1 ng/µL recombinant human PKD1 and 20 µg Syntide-2 substrate peptide in 30 µL kinase buffer (50 mM Tris-HCl, pH 7.5, 4 mM MgCl_2_ and 10 mM β-mercaptoethanol) were incubated with a concentration range (0–100 µM) of test compound. Kinase reactions were initiated with the addition of ATP (50 µM ATP, 1.0 µCi γ-^33^P-ATP; Perkin Elmer, Boston, MA) and incubated for 20 min at 30°C. Twenty-five µL of the reaction was spotted onto Whatman P81 filter paper (Whatman Inc., Clifton, NJ). Filter papers were then washed three times in 0.5% phosphoric acid, air-dried, and counted using an LS6500 multi-purpose scintillation counter (Beckman Coulter, Fullerton, CA). IC_50_ determinations were performed using GraphPad Prism software 5.0. Radiometric PKD2 and PKD3 *in vitro* kinase assays were performed as previously described [18].

For ATP competition studies, recombinant human PKD1 (1 ng/µL) and Syntide-2 substrate peptide (200 ng/µl) in kinase buffer were incubated with a concentration range of test compound. Kinase reactions were initiated with a concentration range of cold ATP (12.5–1000 µM) supplemented with 1.0 µCi γ-^33^P-ATP (Perkin Elmer) and processed as described above. Enzyme inhibition was analyzed and visualized using the Michaelis-Menten and Lineweaver-Burk functions of GraphPad Prism 5.0.

### Western blot analysis

Confirmed PKD1 inhibitors were also evaluated for effects on intracellular PKD1 phosphorylation as previously described [18]. Briefly, prostate cancer cells (LNCaP) were exposed to test compounds. Cells were lysed in buffer (200 mM Tris-HCl, pH 7.4, 100 µM 4-(2-aminoethyl) benzenesulfonyl fluoride, 1 mM EGTA, and 1% Triton X-100) and cell lysates were subjected to SDS-PAGE then electrotransferred to nitrocellulose membranes. Membranes were blocked with 5% nonfat milk in Tris-buffered saline. Membranes were probed with a primary antibody then followed by a secondary anti-mouse or anti-rabbit secondary antibody conjugated to horseradish peroxidase (1∶1000, Bio-Rad). Bands were visualized by the enhanced chemiluminescence Western blotting detection system (Amersham Biosciences). The primary antibodies included *p*-S916-PKD1 antibody (Millipore) and GAPDH (Enzo Life Sciences, Plymouth Meeting, PA).

### HTS data analysis and visualization, statistical analysis and database mining

Primary HTS data analysis and subsequent test compound IC_50_ calculations were performed using ActivityBase (IDBS, Guildford, UK) and CytoMiner (UPDDI, Pittsburgh, PA). Additional data visualization and statistical analysis were performed using GraphPad Prism 5.0 and Spotfire (Somerville, MA). The PubChem database (http://PubChem.ncbi.nim.nih.gov) was mined to determine if the confirmed PKD1 inhibitors registered as confirmed inhibitors in additional *in vitro* and cell-based assays. MetaDrug™, is a systems pharmacology platform specifically designed for evaluating the biological effects of small molecule compounds (GeneGo Inc., St. Joseph, MI). MetaDrug includes a comprehensive chemical and pharmacological database that comprises chemical structures of over 680,000 drugs, metabolites, and xenobiotics along with data on their biological effects from over 1,000,000 *in vitro* pharmacological-binding assays; 1,500,000 *in vitro* functional assays; and 600,000 *in vivo* functional assays.

### Structural similarity, morphological similarity scoring and molecular docking studies

Structural similarity of the confirmed inhibitors was determined using Leadscope (Columbus, OH). Molecular alignment and morphological similarity scoring was performed using Surflex-Sim (Tripos, St. Louis, MO).

Flexible docking studies were performed using Molegro Virtual Docker (MVD, University of Aarhus, Denmark) to evaluate the potential binding of confirmed PKD1 small molecule inhibitors to the conserved ATP binding site [29]. The basis of these docking studies was a new hybrid search algorithm, called guided differential evolution, which combined differential evolution optimization with a cavity prediction algorithm that was dynamically used during the docking process. Briefly, all individual ligands were initialized, evaluated and scored (*E_score_*) according to the fitness function which was the sum of the intermolecular interaction energy between the ligand and the protein and the intramolecular interaction energy of the ligand (eq. 1) [29].

*E*_score_ = *E*_inter_+*E*_intra_, with *E*_inter_ being the ligand-protein interaction energy and *E_intra_* being the internal energy of the ligand (eq. 1)
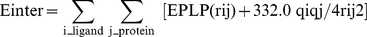


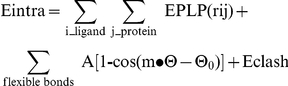

*E*_PLP_ is a piecewise linear potential using two different sets of parameters: one set for approximating the steric van der Waals term between atoms and the other stronger potential for hydrogen bonds. *E*_clash_, assigns a penalty of 1000 if the distance between two atoms (more than two bonds apart) is less than 2.0 Å. Thus, the *E*_clash_ term punishes infeasible ligand conformations. Offspring was created using a weighted difference of the parent solutions, which were randomly selected from the population. If, and only if, the offspring was a better fit would it replace the parent compound. Otherwise, the parent survived and was passed on to the next generation, representing an iteration of the algorithm. The search process was terminated when the number of fitness evaluations exceeded the maximum number of evaluations permitted.

## Results

### Identification of novel small molecule inhibitory chemotypes for protein kinase D using a validated automated assay system

A PKD1 IMAP FP assay was developed and optimized to identify small molecule inhibitors of PKD1 kinase activity. Using this assay system, we screened the LOPAC and PMLSC compound sets (196,425 total compounds) for novel PKD inhibitory chemotypes. The LOPAC set, a collection of 1,280 compounds of known pharmacological actions that impact many cellular processes and includes major drug target categories, had a dual purpose by validating our optimized automated assay system as well as serving as a pilot screen. For the LOPAC screen each compound was evaluated in duplicate at a single concentration (10 µM). Eight known kinase inhibitors were identified as primary actives (≥50% inhibition of control signal) in the PKD1 IMAP FP screen of the LOPAC set (Table S1), including previously described nonspecific PKD1 inhibitors H7, H8 and H89 (our assay MIN control) [30]. The validated HTS assay then was used to screen the available full panel PMLSC diversity-based library which consisted of 196,173 compounds. Similarly, this library was screened at 10 µM. One hundred and nine compounds from the PMLSC library exhibited >50% inhibition of the control signal (for a 0.06% primary hit rate) and advanced to secondary hit confirmation IMAP FP assays. From the LOPAC screening activities, TG003 was prioritized to proceed to secondary confirmation assays as the majority of the other primary active compounds identified were known general kinase inhibitors [31].

### Confirmation of PKD1 small molecule inhibitory chemotypes by in vitro concentration response assays

**Figure 1:**
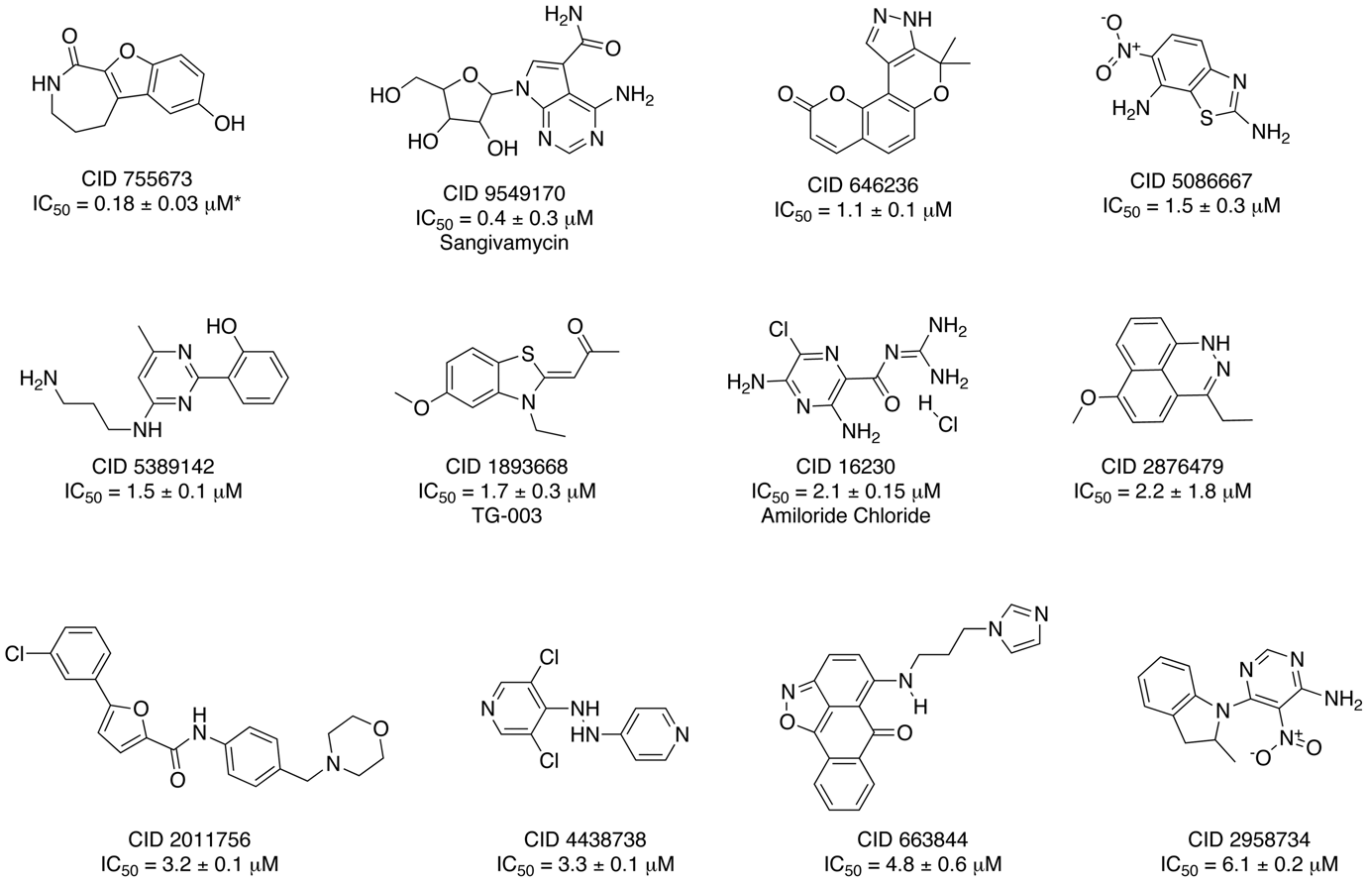
Chemical structures of new PKD1 small molecule inhibitors identified through high throughput screening. New PKD1 inhibitory chemotypes were identified through HTS activities with twelve inhibitory chemotypes confirming in a radiometric PKD1 kinase assay. *In vitro* IC_50_ values ranged from 0.2–6.1 µM. Data are represented as mean ± range or SD of 2–4 independent experiments.

False positive chemotypes were eliminated from the initially identified putative PKD1 inhibitors by repeating the primary single concentration assay followed by a 10-point concentration response assay (data not shown) [32]. Subsequently, twelve prioritized compounds identified from the PMLSC and LOPAC screens were obtained from commercial sources and evaluated in a radiometric PKD1 kinase assay. All 12 chemotypes confirmed as inhibitors of *in vitro* PKD1 kinase activity and ranged in potency from 0.2–6.1 µM. Two compounds exhibited nanomolar activity (*i.e.*, sangivamycin and CID 755673), while the remaining ten compounds clustered with low, single digit micromolar PKD1 inhibitory activity. Structural cluster analysis by Leadscope indicated that all 12 chemotypes were structurally distinct (Figure 1). Of the 12 PKD1 inhibitors, three compounds have previously described pharmacological actions, sangivamycin (CID 9549170), a known PKC inhibitor; amiloride chloride (CID 16230), a sodium channel blocker; and TG-003, a Cdc2-like kinase (Clk) inhibitor (CID 1893668) (Figure 1) [31]. None of the 12 PKD1 inhibitory chemotypes appeared to be redox active at concentration of ≤10 µM based on a previously described H_2_O_2_ generation assay (PubChem AID 878) [33].

### Select novel PKD1 inhibitory chemotypes decrease the phosphorylation of endogenous PKD1 Ser^916^ in LNCaP cells

**Figure 2:**
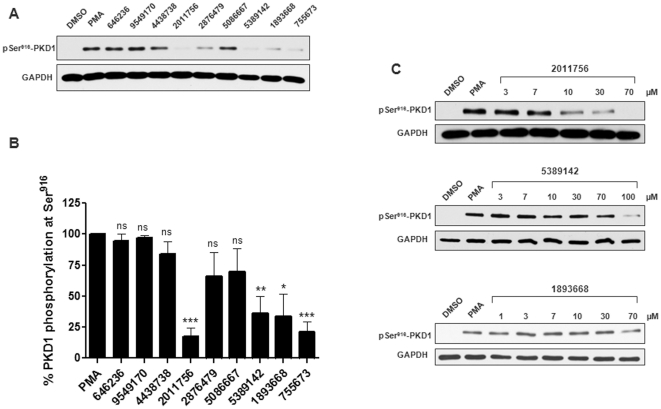
Select, novel PKD1 SMI display inhibition of cellular PKD1 phosphorylation at Ser^916^. Panels A and B, compounds were evaluated initially for PKD1 inhibitory activity at 50 µM in LNCaP cells. Panel C, a representative Western blot shows that four compounds CID 2011756, CID 5389142, CID 1893668 and CID 755673 (control compound), displayed a concentration-dependent inhibition of PMA-induced, endogenous PKD1 activity. Quantification of Western blotting data was determined using densitometry analysis and is graphically represented as mean ± SEM. Data presented are representative of three to four independent experiments. ns, not statistically significant; *, p<0.05; **p<0.01; ***, p<0.001.

Confirmed *in vitro* PKD1 inhibitors were then evaluated for their ability to inhibit the phosphorylation of endogenously expressed PKD1 Ser^916^, an indicator of cellular PKD1 kinase activity. Initially, LNCaP cells were pretreated with a single concentration (*i.e.*, 50 µM) of test compound for 45 min, followed by PMA stimulation at 10 nM for 20 min. Whole cell lysates were immunoblotted for phosphorylated Ser^916^-PKD1 with GAPDH serving as a loading control. As expected, CID 755673 confirmed as a cell active PKD1 inhibitor; however, results showed that three additional chemotypes (*i.e.*, CID 2011756, 5389142 and 1893668) also inhibited the phosphorylation of Ser^916^-PKD1 at levels greater than 50% (Figure 2, panels A and B). Two additional chemotypes, CID 2876479 and 5086667 displayed some cellular inhibition of phospho-Ser^916^-PKD1 activity; however, the inhibition was more variable and not statistically significant. Pretreatment of LNCaP cells with the remaining CIDs 16230, 2958734 and 663844 resulted in little or no inhibition of the phosphorylation of Ser^916^-PKD1 (Figure S1). Cellular EC_50_ values were then determined for the three most potent, cell active PKD1 small molecule inhibitors, CIDs 5389142, 18936668, and 2011756 (Figure 2, panel C). CIDs 5389142 and 1893668 had cellular EC_50_s of 22±3 (n = 3) and 52±14 µM (n = 3), respectively. CID 2011756 was the most potent of the inhibitors with a cellular EC_50_ of 10±0.7 µM (n = 3), an EC_50_ value comparable to that of our previously described benzoxoloazepinolone (*i.e.*, CID 755673).

It was also gratifying that, in general, our novel cell active PKD1 inhibitors appeared to be less promiscuous than PKD1 inhibitors having minimal to no cellular PKD1 inhibitory activity (Table 1). For example, CIDs 755673 and 1893668 display additional confirmed activity in six and seven, respectively, PubChem bioassays while CID 2011756 has confirmed activity in only one additional PubChem bioassay. CID 5389141 has not confirmed in any other PubChem assay. In contrast, the remaining chemotypes registered as confirmed actives in 2–43 additional bioassays (Table 1). Additional chemical similarity searching, focusing on our four novel cell-active PKD1 inhibitory chemotypes, using MetaDrug also supports a comparative lack of promiscuity of these compounds. MetaDrug data mining analyses of CIDs 2011756, 5389142 and 755673 did not reveal any additional target-based inhibitory activity of those specific parental structures (Figure S2). However, several compounds structurally similar (70–75%) to CID 755673, showed inhibitory activity against NR2B, alpha-2C-1 adrenergic receptor, and melatonin receptors 1A and 1B (Table S2). As expected, MetaDrug data mining for TG003 revealed its Clk1 inhibitory activity (Table S2). Moreover, we counter screened CIDs 5389142 and 2011756 in IMAP-based CDK7, AKT and PLK1 assays, and found no kinase inhibition at concentrations up to 50 µM. Taken together, these analyses suggest a low degree of promiscuity of our novel cell active PKD1 inhibitors.

**Table 1:** Comparison of confirmed PKD1 small molecule inhibitors.

PubChem CID	Confirmed actives/Pubchem AIDS^1^	Tanimoto and Morphological Similarity Scores (T/MSS)(ATP)	Mechanism of Action
**Cell Active**			
755673	6/558 (1.1%)	0.42/0.45	Non-ATP competitive
2011756	1/444 (0.2%)	0.46/0.45	ATP competitive
5389142	0/550 (0.0%)	0.43/0.62	ATP competitive
1893668	7/632 (1.1%)	0.37/0.53	NT
**Minimal**			
5086667	03/423 (0.7%)	0.44/0.60	ATP competitive
16230	10/710 (1.4%)	0.40/0.63	NT
646236	5/527 (1.0%)	0.32/0.60	ATP competitive
663844	43/663 (6.5%)	0.46/0.53	NT
4438738	2/404 (0.5%)	0.26/0.61	ATP competitive
2876479	20/431 (4.6%)	0.31/0.53	ATP competitive
9549170	17/430 (4.0%)	0.79/0.60	ATP competitive
2958734	7/351 (2.0%)	0.47/0.52	ATP competitive

1 Pubchem checked 4/10/11. PKD1 confirmation AID not included as a confirmed active.

### New PKD1 inhibitory chemotypes are competitive with ATP

**Figure 3:**
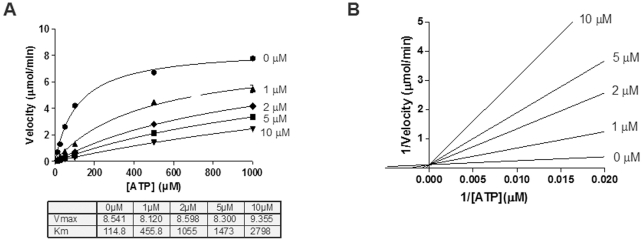
Confirmed novel PKD1 inhibitors display competitive activity with respect to ATP. Select PKD1 inhibitors were evaluated to determine if they were mixed, competitive or non-competitive inhibitors of ATP. Panels A and B, representative Michaelis-Menten and Lineweaver-Burk plots of CID 5086667. This compound was found to be an ATP-competitive inhibitor. Data presented were representative of three independent experiments.

To expedite the characterization of our PKD1 inhibitors, we evaluated chemical structures computationally for similarities to ATP using both Tanimoto score (TS) and morphological similarity score (MSS) methodologies. As expected sangivamycin, a known ATP competitive inhibitor, was predicted to be moderately to strongly similar to ATP depending on the scoring system. Moreover, CID 755673, a non-ATP competitive inhibitor, was predicted to be weakly similar to ATP structure with both the TS and MSS methodologies. Less congruency was detected between the two predictive methodologies for the remaining 10 novel PKD1 inhibitory compounds. However, with MSS more compounds displayed a moderate similarity (MSS = ∼0.6) to ATP, suggesting that our PKD1 inhibitors may be biased towards ATP competitive scaffolds. Experimentally, these MSS predictions were borne out with 8 of 8 tested compounds being ATP competitive (Table 2). Figure 3 shows ATP competition data from a representative compound, CID 5086667. Of the total nine compounds evaluated for mechanism of action, eight compounds were determined to be ATP competitive while the remaining compound, CID 755673, was previously determined to be non-ATP competitive [18]. We elected not to evaluate CID 16230, 1893668 and 663844 based on their promiscuity and/or already existing data on mechanism of action (*i.e.*, CID 1893668 – ATP competitive) [31]. Moreover, preliminary *in vitro* data suggest that our most potent cell active PKD1 inhibitor, CID 2011756, has pan-PKD inhibitory effects (PKD2 IC_50_ = 0.6±0.1; PKD3 IC_50_ = 0.7±0.2 µM) with similar, albeit not identical, potencies which may be expected for an ATP competitive inhibitor.

**Table 2:** Structural dissimilarity of small molecule inhibitors with cellular PKD1 inhibitory activity.

	BKPDi		CRT5		PubChem CID 755673	
PubChem CID	TS^2^	MSS^3^	TS	MSS	TS	MSS
2011756	0.55	0.60	0.47	0.57	0.57	0.40
5389142	0.47	0.57	0.44	0.59	0.46	0.15
755673	0.39	0.55	0.42	0.53	1.0	1.0
1893668	0.26	0.39	0.45	0.41	0.42	0.40

2 –Tanimoto Score;

3 – Morphological Similarity Score.

### The cell-active PKD1 inhibitors are diverse in structure

As noted, Leadscope analysis indicated that our 12 PKD1 small molecule inhibitors were structurally distinct with respect to each other, highlighting the diversity of our identified chemotypes. Thus, we next evaluated our three novel cell-active PKD1 inhibitory compounds for structural similarity to BKPDi, CRT5 and CID 755673 (Table 2). Data derived from both TS and MSS analyses indicated that our new PKD1 small molecule inhibitors displayed weak (CID 1893668) to moderate (CIDs 2011756 and 5389142) structural similarity to the previously aforementioned cell-active PKD1 inhibitors. Thus, it would appear we have increased the structural diversity of cell-active PKD1 small molecule inhibitors, encouraging the development of more potent inhibitors using these parental structures in future structure activity relationship iterations.

### Molecular docking of BKPDi, CID 2011756 and CID 5389142 to a conserved ATP binding cleft suggests CID 5389142 may afford opportunities for structural modification to enhance its physicochemical properties

**Figure 4:**
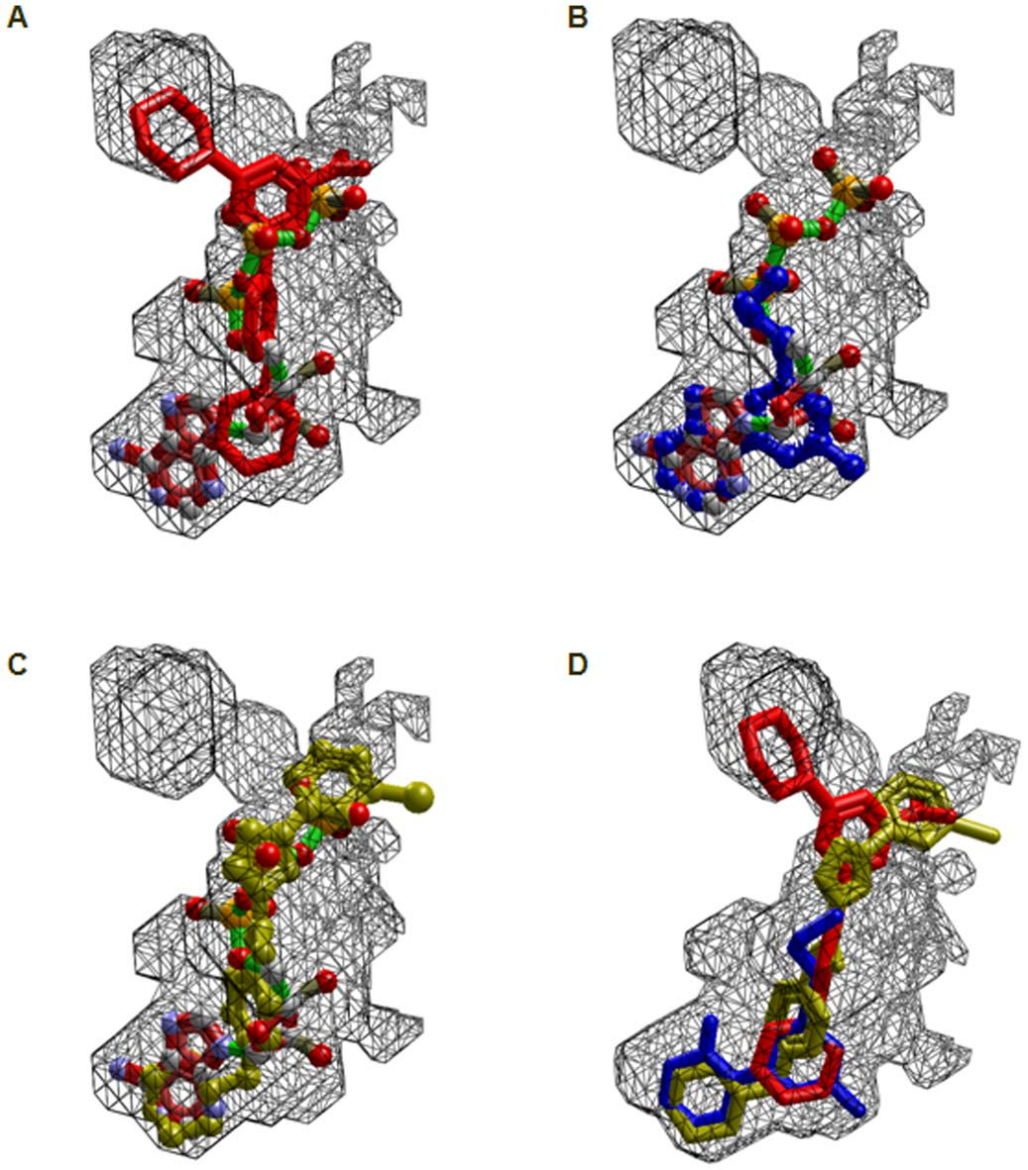
Docking simulations of select cellular active PKD1 small molecule inhibitors in a conserved PKA catalytic core. Panel A shows the binding pose of BPKDi (red), a cell-based PKD inhibitor, with a known mechanism of action (*i.e.*, ATP competitive) and ATP (ball-and-stick representation and colored by atom type). Panels B and C depict our two cellular PKD1 small molecule inhibitors and ATP docked to the conserved catalytic domain (Panel B, CID 5389142 (blue); Panel C, CID 2011756). Panel D displays all three cellular PKD1 small molecule inhibitors (CID 5389142-blue, CID 2011756-yellow and BPKDi-red) docked to the conserved ATP binding domain.

To help characterize how our novel cell-active PKD1 inhibitors are differentiated from previously described PKD1 small molecule inhibitors, we performed docking studies using Molegro Virtual Docker (MVD). Predictions for interactions between the conserved ATP binding site of PKA with the compounds described in Table 3 were evaluated [17]. We first validated our system by removing ATP from the binding site and docked using MVD to see if the docking pose was close to the position determined through X-ray crystallography. All side-chain residues in the ATP-binding site were set flexible. The dock score of −176.75, and the low root mean square deviation (RMSD) between the X-ray pose and best docked pose (*i.e.*, 0.45 Å) suggested that the docking protocol was accurate. Figure S2 displays the superposition between the docked pose and the X-ray pose. BKPDi, CID 2011756 and CID 5389142 were then docked (n = 10 independent runs) into the conserved ATP binding site, poses were generated and then ranked by energy. The lowest energy pose of the 10 poses per compound was selected for visualization (Figure 4). Data indicated that BKPDi (Figure 4, panel A) and CID 2011756 (Figure 4, panel C) bind tightly, although not identically, in the conserved ATP binding cleft with a dock score of −138.68 and −136.32, respectively (Table 3). Not surprisingly, CID 5389142, a smaller compound, displayed a relatively low dock score of −91.51 (Figure 4, panel B and Table 3), suggesting that the smaller size of the compound may afford more opportunities for structural enhancement.

**Table 3:** Binding energies of PKD1 inhibitors with an ATP binding domain.

PubChem CID	Dock Score	Mechanism of Action
ATP	−176.75	N/A
BKPDi	−138.32	ATP
5389142	−91.51	ATP
2011756	−136.32	ATP

## Discussion

In the study presented here, we describe an *in vitro* HTS campaign designed to identify novel inhibitory chemotypes of PKD1 kinase activity. Using this HTS assay system, we screened two distinct compound libraries, the LOPAC and the contemporary PMLSC library, and identified and confirmed the PKD1 inhibitory activity of 12 chemotypes. CID 755673 was previously reported by us as a potent, cell-active non-ATP competitive pan-PKD inhibitor and has been the focus of an analogue development strategy, which has successfully yielded multiple compounds with increased *in vitro* and cellular PKD inhibitory activity. The remaining 11 compounds identified from our screening efforts are now described for the first time. Of these new chemotypes, three additional compounds (*i.e.*, CIDs 2011756, 5389142 and 1893668) exhibited cellular activity while the remaining 8 compounds displayed minimal or no cellular activity. These novel PKD1 inhibitory chemotypes appeared to be ATP-competitive and all are structurally unrelated. Moreover, the new cell-active PKD1 inhibitory chemotypes were structurally distinctive from the previously reported inhibitors BKPDi, CRT5 and CID 755673 that also have cellular activity for PKD1. Thus, we successfully expanded the diversity of the pool of available PKD1 small molecule inhibitors, which lay the foundation for the synthesis of more potent, cell-active PKD1 inhibitors.

One significant advantage of our PKD1 small molecule inhibitor studies is that all of our confirmed chemotypes described here are available through commercial sources, allowing for an immediate accessibility to the general scientific community. This significant advantage is, of course, due to the types of libraries (*i.e.*, LOPAC and PMLSC sets) that were selected and interrogated for PKD1 inhibitory chemotypes. A ramification of the choice of libraries is that structural information is readily and publicly available for compounds, allowing for multiple groups to explore the reported biological and chemical attributes. Moreover, as our compounds are part of the PubChem database (http://PubChem.ncbi.nlm.nih.gov/) they have been evaluated in hundreds of *in vitro* and cellular screening assays, providing insight of the potential specificity or promiscuity of a particular chemotype. However, although data-mining PubChem (or any other database) is an effective resource, it should be part of a more extensive secondary screening strategy. Observations derived from PubChem data-mining should be independently and rigorously evaluated as the data posted is limited to an early stage confirmation.

Potent, general kinase inhibitors, such as H-89, H-7, H-8 and Gö6976 have been available as PKD1 inhibitors for over 15 years [30], [34]. Due to the historic lack of PKD1 inhibitors, they became valuable and effective research tools by necessity. However, they are also multi-target kinase inhibitors [35], [36], [37]. Thus, we cannot specifically knockdown PKD1 activity in cellular signaling pathways using these compounds without directly impacting other kinases. Only in the past two to three years have we seen reports appear in the literature of potent and specific PKD1 small molecule inhibitors [18], [22], [23], [24], [25], [26], [27], [28]. These PKD1 inhibitors are structurally diverse and have cellular activity, but vary in the accessibility of structural information, commercial availability, oral bioavailability, and reported characterization. Based on the unique aspects of CID 755673 (*i.e.*, potency, mechanism of action) we prioritized it for analogue development. However, the subsequent identification of additional potent and specific PKD1 inhibitors prompted us to revisit our other eleven chemotypes that displayed *in vitro* PKD1 inhibitory activity. As a result of our efforts, we have now identified multiple compounds with cellular PKD1 inhibitory activity, adding to the structural diversity of reported PKD1 small molecule inhibitors and allowing for additional analogue development.

Historically, the paucity of PKD1 inhibitors has been a hindrance to PKD1 studies; however, with multiple reports of PKD1 small molecule inhibitory chemotypes, this situation appears to be at least partially resolved. Nonetheless, the lack of PKD1 structural information continues to be a challenge for PKD1 research by limiting our ability to use structure-based approaches to design more potent and specific small molecule inhibitors. Thus, we utilized structural information of the highly conserved kinase active site cleft to help characterize the predicted binding profile of several ATP-competitive PKD1 small molecule inhibitors with cellular activity. Specifically, we based our strategy on the work of Thompson *et al* [27] who demonstrated that the kinase active site cleft is highly conserved among multiple kinase families as a single, contiguous, irregularly shaped cleft similar in size and geometry. By docking specific PKD1 small molecule inhibitors to this conserved active site cleft we observed that the interaction with the kinase active site cleft may assist with the identification of compounds for structural modification. Moreover, this suggests that in the absence of structural data for PKD1 we may be able to capitalize on available structural information of highly conserved protein domains or sites to assist with the design of PKD1 small molecule inhibitors.

## Supporting Information

Figure S1 **Lack of potent cell-based PKD1 inhibitory activity of CIDs 16230, 2958734 and 663844.** The remaining three *in vitro* active PKD1 inhibitory compounds were screened for LNCaP cell-based activity at 50 µM. Panel A, Representative image of a cell lysate Western blot. Panel B, Quantification of a Western blots (mean ± range) (n = 2). One compound, CID 16230, exhibited less than 50% inhibitory activity of PKD1. Due to its low activity, the cellular EC_50_ of CID 16230 could not be accurately determined.
(PPTX)

Figure S2 3D Structure of the cAMP-dependent protein kinase A (PKA) (PDB ID:3fjq) and the ATP binding pocket.
(PPTX)

Table S1 Actives identified from two independent evaluations of the LOPAC set.
(PPTX)

Table S2 MetaDrug data mining for our four novel PKD1 inhibitors with cell-based activity.
(PPTX)
